# Deliberate self-harm in adolescents screening positive for attention-deficit / hyperactivity disorder: a population-based study

**DOI:** 10.1186/s12888-024-06008-3

**Published:** 2024-08-19

**Authors:** Amalie Austgulen, Maj-Britt Posserud, Mari Hysing, Jan Haavik, Astri J. Lundervold

**Affiliations:** 1 Department of Biomedicine, Faculty of Medicine, University of Bergen, Bergen, Norway; 2 Department of Child and Adolescent Psychiatry, Division of Psychiatry, Haukeland University Hospital, Bergen, Norway; 3 Department of Clinical Medicine, Faculty of Medicine and Dentistry, University of Bergen, Bergen, Norway; 4 Department of Psychosocial Science, Faculty of Psychology, University of Bergen, Bergen, Norway; 5 Regional Centre for Child and Youth Mental Health and Child Welfare, NORCE Norwegian Research Centre, Bergen, Norway; 6 Division of Psychiatry, Haukeland University Hospital, Bergen, Norway; 7 Department of Biological and Medical Psychology, University of Bergen, Bergen, Norway

**Keywords:** ADHD, Adolescence, Attention deficit hyperactivity disorder, Deliberate self-harm, Self-injury, Self-harm

## Abstract

**Background:**

Adolescents with attention-deficit / hyperactivity disorder (ADHD) have an increased risk of self-harm. The risk of self-harm among adolescents who display an elevated level of ADHD symptoms, but without a formal diagnosis, is not well-studied and understood.

**Objective:**

To investigate the relationship between self-reported symptoms of ADHD and self-harm in a population-based sample of adolescents.

**Methods:**

Adolescents in the population-based youth@hordaland study were invited to complete the Adult ADHD Self-Report Scale (ASRS) and the Short Mood and Feelings Questionnaire (SMFQ). They were asked whether they ever deliberately have taken an overdose or tried to harm themselves on purpose, once or multiple times, defined according to the code used in the Child and Adolescent Self-harm in Europe (CASE) Study. Adolescents reporting severe problems on ≥ four of six selected items on the ASRS-v 1.1 screener were defined as ADHD-screen positive (ADHD-SC+), and the remaining sample as ADHD-screen negative (ADHD-SC-). SMFQ score ≥ 12 was used to define a high level of depressive symptoms.

**Results:**

A total of 9692 adolescents (mean age 17.4 years, 53.1% females) participated in the study, of which 2390 (24.7%) screened positive on the ASRS. ADHD-SC+ adolescents engaged in self-harm more often than the ADHD-SC- group (14.6% vs. 5.4%, OR = 3.02, 95%CI [2.57–3.24]). This remained significant after adjustment for demographic variables, SMFQ score ≥ 12, symptoms of conduct disorder and familial history of self-harm and suicide attempts (OR = 1.58, 95%CI [1.31–1.89]). They were also more likely to report an overdose as their method of self-harm (OR = 1.52, 95%CI [1.05–2.23]). Within the ADHD-SC+ group female sex, high levels of inattention and hyperactivity/impulsivity symptoms, SMFQ score ≥ 12, symptoms indicating conduct disorder and familial history of self-harm and suicide attempts increased the likelihood of engaging in deliberate self-harm.

**Conclusion:**

Adolescents who screened positive for ADHD had increased risk of engaging in self-harm. Clinicians should consider the increased risk of such engagement in adolescents who present with high level of ADHD symptoms, even in the absence of a clinical ADHD diagnosis.

**Supplementary Information:**

The online version contains supplementary material available at 10.1186/s12888-024-06008-3.

## Introduction

Self-harming behaviors are common among adolescents and have been shown to increase future risk of suicide [1–4]. The risk of self-harm is notably elevated among persons with a diagnosis of attention-deficit/hyperactivity disorder (ADHD) [1]. Less is known about the risk of self-harm in adolescents who display elevated levels of ADHD symptoms in the general population.

ADHD is a childhood-onset neurodevelopmental disorder, with symptoms that often continue throughout adolescence and adulthood. In Norway, approximately 4% of adolescents at age 16 are diagnosed with ADHD, with the highest prevalence in boys (5.5%) [5]. Core symptoms include inattention, hyperactivity, and impulsivity [6], but individuals with ADHD also frequently struggle with emotion regulation [7–9], high levels of stress, poor executive functioning [10, 11], and comorbid psychiatric disorders [12]. Together, this may lead to challenges affecting quality of life, academic and occupational functioning, and social relationships [13–17]. Individuals with ADHD are also at increased risk of accidents and injuries [18], including deliberate self-harm.

Self-harm is defined as intentional self-inflicted destruction of one’s body, and is often classified as non-suicidal self-injury (NSSI) if there is no suicidal intent present [19]. The estimated prevalence is 16.2% in a Norwegian population-based sample of adolescents, with similar prevalence rates reported worldwide, making self-harm a major public health problem in this age group [20, 21]. In recent years, several reviews and large cohort studies have established a positive association between ADHD and both self-harm and suicide attempts [1, 22–26]. In population-based studies, adolescents and young adults with ADHD face a heightened risk of self-harm when compared to their peers without the diagnosis [27–29]. Furthermore, a study investigating a nationally representative sample of Australian youths found that even adolescents with subthreshold ADHD had an increased risk of NSSI [30]. Previous studies have proposed several important risk factors in the association between ADHD and both self-harm and suicide, including higher levels of ADHD symptoms, female sex, and psychiatric comorbidities, such as depression, bipolar disorder, and substance use disorder [31–44].

While ADHD is more commonly diagnosed in males than females, there is conflicting evidence regarding the role of sex differences in the risk of self-harm. A clinical study investigating psychiatric inpatient adolescents found that an ADHD diagnosis was associated with a high likelihood of NSSI, especially in girls [45]. In a case series analyzing adolescents presenting with self-harm at emergency departments, hyperactivity and emotional problems, as measured by the Strengths and Difficulties Questionnaire (SDQ), were found to be significantly higher than in a reference group. Since the sample consisted of 78% females, ADHD symptoms were suggested to be a potential mechanism of recurrent self-harm in female adolescents [46]. Another study found no significant effects of sex on self-harm in a clinical sample of Canadian children and adolescents with ADHD [47]. This highlights the undetermined significance of ADHD symptoms, which could have potentially different clinical implications in relation to self-harm in males and females.

There is some conflicting evidence regarding the relative importance of each symptom domain of ADHD in relation to self-harm. Recently, it was observed that inattention in childhood was closely associated with NSSI at 15 years of age, which was not the case for childhood impulsivity or hyperactivity [48]. This was also explored in the Berkeley Girls with ADHD Longitudinal Study (BGALS), where both inattention and hyperactivity / impulsivity symptoms were more severe in adolescent girls who had a history of NSSI [32]. On the other hand, a cross-sectional study investigating a clinical sample of 1006 Canadian children and youth indicated that a selection of hyperactivity-impulsivity symptoms, but not inattention, were associated with NSSI [49]. In adults with ADHD who presented with self-harm at Swedish hospitals, impulsivity was not found to be a significant predictor of self-harm, but this was after adjustments for a clinical diagnosis of depression and emotionally unstable personality disorder, sex, and age [42].

Self-harm in adolescents is often associated with higher severity levels of depressive symptoms [4, 21], and depression is a common comorbid disorder in individuals with ADHD [37, 39, 40, 50]. A study utilizing population-based data from the youth@hordaland study found that each severe ADHD symptom, as reported on the Adult ADHD Self-Report Scale (ASRS), significantly contributed to an increase in the score of depressive symptoms, measured by the Short Mood and Feelings Questionnaire (SMFQ) [51]. More than 20% of those who were defined as depressed reported six or more symptoms of inattentiveness, suggesting a strong link between a diagnosis of ADHD and depression, particularly in females. In males, symptoms of hyperactivity and impulsivity have been suggested to be closely associated with externalizing disorders through shared developmental pathways, predisposing vulnerabilities, and environmental influences [52].

The role of comorbidities in predicting self-harm in adolescents is still not fully understood, but several large cohort studies have shown that the association between ADHD and self-harm remains statistically significant even after adjustments for the presence of comorbid psychiatric disorders [39, 40, 42, 50, 53]. However, other studies have reported conflicting results, as symptoms of comorbid conditions in both sexes have been found to fully mediate the relationship between symptoms of ADHD and the presence of NSSI [45]. Additionally, findings from a population-based study in Denmark indicate that individuals with ADHD have a higher risk of suicidal behavior when a family history of psychiatric disorders or suicidal behaviors is present [37]. This highlights the significance of considering comorbid symptoms not only in individuals with ADHD, but also in their family members.

In previous literature, the association between ADHD and suicidal behavior has been studied extensively [22, 23, 25, 26, 54]. The majority of these studies have included clinical samples. However, there is still a need for more information regarding self-harm in adolescents who exhibit elevated levels of ADHD symptoms, especially on a population-based level. Since both ADHD symptoms and self-harm are especially prevalent in this age group, more information on their interaction could improve the understanding of this relationship and possible implications.

### Objective

The aim of the present study was to investigate the relationship between self-reported symptoms of ADHD and self-harm in a population-based sample of adolescents. First, we sought to estimate the prevalence of adolescents engaged in self-harm across ADHD screening status. Furthermore, we investigated how factors such as sex, inattentive and hyperactivity/impulsivity symptoms, symptoms of depression and conduct disorder, as well as familial history of self-harm and suicide attempts, affected the likelihood of engaging in self-harm, both once and multiple times, in a non-clinical sample of adolescents who screen positive for ADHD.

## Methods

### Study design

The present study included data from the cross-sectional population-based study youth@hordaland. The overall aim of the youth@hordaland study was to gather information about mental health problems, lifestyle factors, and service use among adolescents living in Hordaland County in Norway.

### Setting

All adolescents born between 1993 and 1995, and all students attending upper secondary education between January and May 2012 who were living in Hordaland County in Norway, were invited to participate in the study (19 439). They received information by email, followed by an SMS reminder. All upper secondary schools in the county participated, and the adolescents were allocated time during regular school hours to complete the electronic questionnaire. A teacher was present to organize the data collection and ensure confidentiality.

For the adolescents who were not at school during the allocated time, the questionnaires could be completed at other times during the study period, and some schools arranged new days for catch-up. Those who were in hospitals or institutions were also invited to participate, and arrangements were made to make participation possible. Adolescents not in schools received information by postal mail to their home addresses.

### Ethics

The study was approved by the Regional Committee for Medical and Health Research Ethics (REC) in Western Norway. All adolescents consented to participation in the current study, in accordance with Norwegian regulations stating that adolescents aged 16 and older can make decisions regarding their own health, including participation in health studies. The parents or guardians received written information about the study in advance.

### Variables

#### Age and biological sex

Biological sex and date of birth were identified through the personal identification number in the Norwegian National Population Register. We use the term biological sex when referring to males and females in the present study, though the identified gender of the participants may vary. The age at completion was defined by calculating the time interval between the date of participation and the date of birth.

#### Socioeconomic status (SES)

SES was assessed by adolescent report of parental education for the mother and father, with the response options: “Primary school or similarly”, “Secondary school, vocational”, “Secondary school, general”, “College or university, less than 4 years”, “College or university, more than 4 years” and “do not know”. The two secondary school categories were combined, as well as the categories regarding college or university.

#### Symptoms of ADHD

The Adult ADHD Self-Report Scale (ASRS) was used to assess the presence and severity of ADHD symptoms [55]. This questionnaire is intended for use in adults above the age of 18 but has also been validated in samples of adolescents [56, 57]. It consists of 18 items, with nine items assessing symptoms of hyperactivity/impulsivity (HI) and nine items assessing inattention (IN). The response options are “Never”, “Rarely”, “Sometimes”, “Often” and “Very often”, with scores from 0 to 4. The ASRS has a high internal consistency and has been validated in population-based studies [58].

The first six questions in the ASRS constitute the screener version of the questionnaire, ASRS v 1.1 Screener [55]. Answers “Often” and “Very often”, as well as “Sometimes” on items 1–3, are defined as symptoms highly consistent with ADHD [55]. A score of four or more is indicative of a positive screening for ADHD [58, 59]. This cut-off was used in the present study, with those scoring above being defined as ADHD-screen positive (ADHD-SC+), and the remaining sample as ADHD-screen negative (ADHD-SC-).

In the 18-item ASRS Symptom Checklist (ASRS-18), IN symptoms were defined as severe if reported to be present “Often” or “Very often” on items 1–4 and 7–11, with the addition of “Sometimes” on items 1–3 and 9. Similarly, HI symptoms were defined as severe if participants responded “Often” or “Very often” on items 5–6 and 12–18, with the addition of “Sometimes” on items 12, 16 and 18 [55]. The total number of symptoms at this level was calculated separately for the IN and the HI subscale (0–9) and used to define symptom severity.

The *Diagnostic and Statistical Manual of Mental Disorders*, fifth edition (DSM-5) criteria for ADHD states that children and adolescents under the age of 17 are defined as having high levels of IN or HI if they have 6 or more symptoms of either symptom dimension. For adolescents and adults who are 17 and older, 5 or more symptoms are defined as sufficient. Scores above these cut-offs were used to define a high level of IN or HI symptoms and were applied to select the most affected adolescents in terms of ADHD symptoms.

#### Self-reported ADHD, ADD, and problems with concentration

Participants were asked whether they had received a diagnosis of either ADHD, attention-deficit disorder (ADD), or problems with concentration, not otherwise specified, by a clinical professional.

#### Symptoms of depression

Symptoms of depression were assessed by the Short Version of the Mood and Feelings Questionnaire (SMFQ) [60], which consists of 13 items. The items assess the presence of emotional and cognitive symptoms associated with depression experienced by an individual in the past two weeks, rated on a 3-point Likert scale. SMFQ has shown good psychometric properties and high internal consistency between items in population-based studies [61–64], and has been validated in a study including a sample from youth@hordaland [65].

The total SMFQ score ranges from 0 to 26. In a study examining the reliability and validity of the original and short version of MFQ in adolescents, the optimal cut-off value for differentiating depressed from nondepressed cases was ≥ 12 [66]. This cut-off has also been suggested for young adults [67], while other studies have favored a cut-off score of 11 in this age group [68–70]. In the current study, an SMFQ score of 12 and above was used to dichotomize the adolescents into “low/medium level of depressive symptoms” and “high level of depressive symptoms”.

#### Symptoms of conduct problems

The Youth Conduct Disorder (YCD) scale was used to assess symptoms of conduct problems. This questionnaire is a part of the Diagnostic Interview Schedule for Children Predictive Scales (DPS), which is shown to identify adolescents with a high probability of meeting the diagnostic criteria of conduct disorder [71]. It consists of 8 items covering behaviors such as shoplifting, school expulsion, theft from others, animal cruelty and vandalizing or breaking into the property of others. The response options are “yes” and “no”. In the present study, responses on YCD were dichotomized to having no symptoms of conduct disorder (total score of 0) and presence of conduct problems (total score of 1 or above).

#### Self-harm in participants

To assess whether the adolescents had engaged in self-harm, they were asked the following question: “Have you ever deliberately taken an overdose (e. g., pills or other medication) or tried to harm yourself in some other way (such as cut yourself)?”, which is an item included in the Child and Adolescent Self-harm in Europe Study (CASE) [72]. If participants answered “Yes”, they were asked to complete the following item: “Describe what you did to yourself on that occasion. Please give as much detail as you can - for example, the name of the drug taken in an overdose.” If they had engaged in self-harm more than once, they were asked to report the last time they had harmed themselves.

After the data collection was finished, two coders classified all “yes” answers into “self-harm case” (SH-case), “not SH-case” and “no information on self-harm”. This was done according to the CASE guidelines, defining self-harm as an: “act with a non-fatal outcome in which an individual deliberately did one or more of the following: initiated behavior (e.g., self-cutting, jumping from a height), which they intended to cause the self-harm; ingested a substance in excess of the prescribed or generally recognized therapeutic dose; ingested a non-ingestible substance or object.”. Frequency of self-harm was recorded and coded as follows: “none”, “once”, “two or more times” [4].

In the present study, all participants with answers classifying them as SH-cases were determined to be valid cases, while those who were classified as “not SH-case” and “no information on self-harm” were included in a non-case group together with those who answered “no” on the item assessing self-harm (no-case). Those without any data on self-harm were removed from the sample before the conduction of the statistical analyses. When coding the 908 adolescents who answered yes to having harmed themselves or taken an overdose, 35 (3.9%) were defined as not valid cases of SH from the description given, while 122 (13.4%) adolescents did not give enough information to correctly classify the case according to the CASE guidelines. All SH cases were also coded according to the method used to define self-harm. Due to a limited number of cases using other forms of self-harming methods, only adolescents reporting overdose and/or self-cutting were investigated in this study.

#### Self-harm in family members

Participants were asked: “Have someone in your family ever tried to take their own life or harm themselves on purpose?”, with the response alternatives “No”, “Yes, more than a year ago”, and “Yes, lately”, taken from the CASE study [72]. The last two categories were combined in statistical analyses.

### Statistical analyses

The data was analyzed using *R* (version 4.1.3) [73]. First, we investigated differences in characteristics based on the screening status of the adolescents. Afterward, the association between ADHD screening status and self-harm was explored. We conducted a logistic regression model, with ADHD screening status as the exposure variable, and SH-case versus no-case as the outcome variable. The model was adjusted for age, sex (with male as reference), parents’ level of education (with primary school as reference), SMFQ ≥ 12, YCD ≥ 1, and the familial history of self-harm or suicide attempts. Only participants with complete answers on all measures were included in this model. To investigate differences in characteristics of self-harm, odds ratios (OR) were estimated for those who were ADHD-SC+, with ADHD-SC- as reference. Within the ADHD-SC + group, the same method for estimating ORs was used to explore differences in characteristics between SH cases and non-cases. This was done utilizing the *R* packages *finalfit* and *knitr* [74–77].

## Results

### Sample

**Fig. 1:**
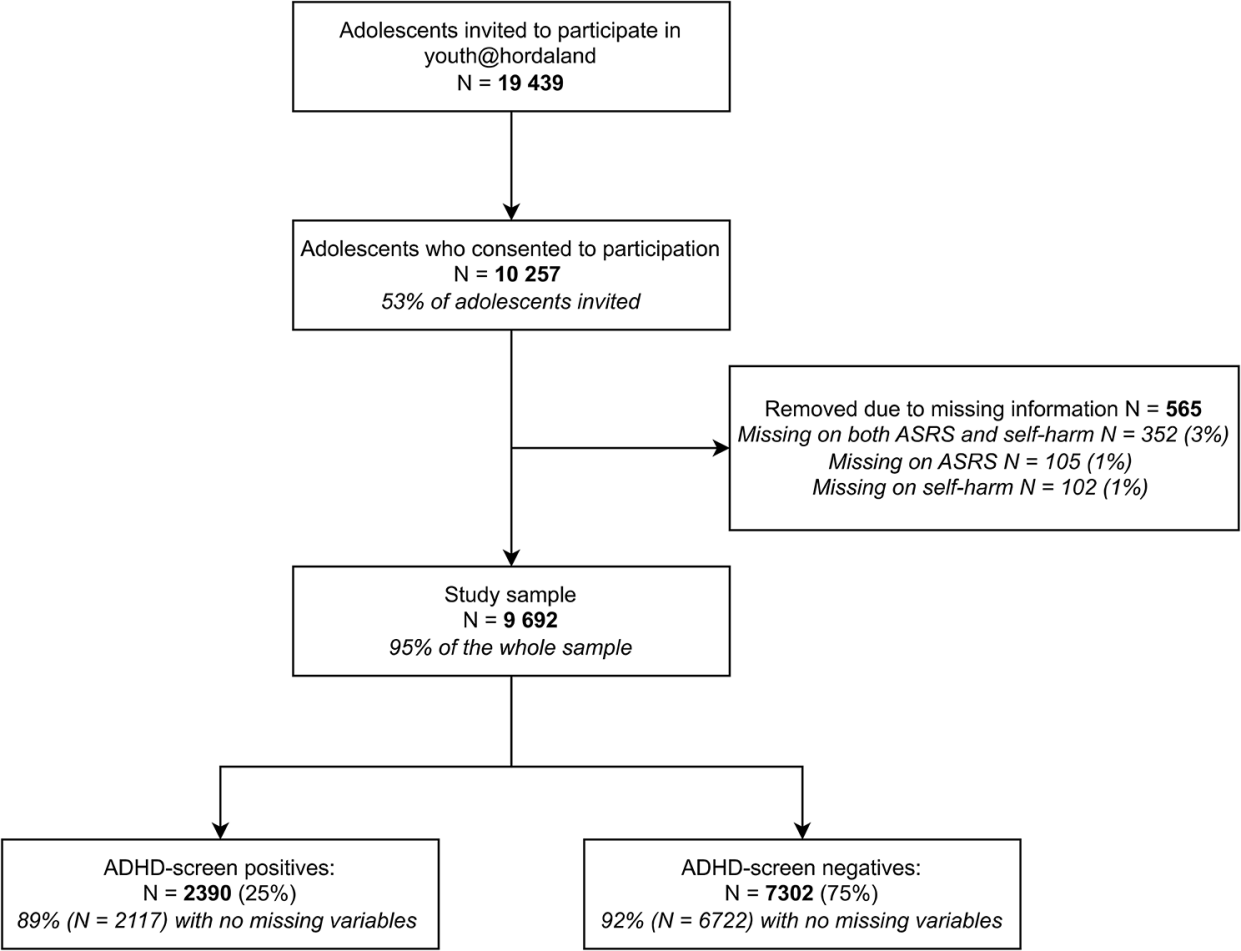
Overview of participants included in the study. ADHD: Attention-deficit / hyperactivity disorder, ASRS: Adult ADHD Self-Report Scale

Out of 19 439 invited adolescents, 10 257 (53%) consented to participate in the study. Among those, 460 (4.5%) adolescents did not complete the ASRS screener version, while 457 (4.5%) did not present complete data on the presence of self-harm. Of those, 352 adolescents had missing data on both measures (Fig. 1). A total of 565 adolescents were removed, constituting the majority of missing variables in the data set (data not shown). The final study sample included 9 692 adolescents, with a mean age of 17.4 years (age range 16–19 years) and comprised of 5165 (53%) females.

When investigating the study sample, 2390 (24.7%) adolescents screened positive on the ASRS-v.1.1 and were categorized as ADHD-SC+. The remaining adolescents (*N* = 7302) were defined as ADHD-SC- (Fig. 1).

A diagnosis of ADHD was reported by 127 adolescents, while 55 reported having attention-deficit disorder (ADD) and 7 reported problems with concentration, not otherwise specified. Out of the 127 adolescents who reported ADHD, 67 (53%) screened positive on the ASRS. This was also the case for 35 (64%) of the adolescents who reported ADD, and 7 (100%) of those who reported problems with concentration.

### Descriptive characteristics

The descriptive characteristics of the sample are presented in Table 1.

Among adolescents who engaged in self-harm (*N* = 745), there were a higher proportion of females (82% vs. 51%, *p* < 0.001), a positive ADHD screening status (47% vs. 23%, *p* < 0.001), and a score of 12 or more on the SMFQ (52% vs. 13%, *p* < 0.001), when compared to adolescents who reported no previous self-harm.

Females who screened positive for ADHD were more likely to report higher levels of IN symptoms (OR 1.56, 95%CI [1.30–1.88]) as well as a high SMFQ score (OR 2.45, 95%CI [2.09–2.88]) compared to their male counterparts. Additionally, ADHD-SC+ females were less likely to report symptoms of conduct disorder (OR 0.67, 95% [0.59–0.76]) than ADHD-SC+ males.

**Table 1:** Baseline characteristics of ADHD screen positive and negative adolescents

	ADHD-SC+ (*N* = 2390)	ADHD-SC- (*N* = 7302)
**Age** at completion ^1^	17.5 +/- 0.8	17.4 +/- 0.8
**Sex**		
Female	1 423 (60%)	3 742 (51%)
Male	967 (40%)	3 578 (49%)
**Mothers’ education**		
Primary school (10 years or less)	214 (9%)	531 (7%)
Secondary school (11–13 years)	752 (32%)	2 277 (31%)
Higher education (13 + years)	777 (33%)	2 774 (38%)
Don’t know	629 (27%)	1671 (23%)
**Fathers’ education**		
Primary school (10 years or less)	231 (10%)	527 (7%)
Secondary school (11–13 years)	787 (33%)	2 548 (35%)
Higher education (13 + years)	648 (31%)	2 424 (33%)
Don’t know	350 (15%)	1 737 (24%)
**ASRS score**		
Mean score (+/- SD)	48.3 +/- 11.1	29.5 +/- 10.8
Number of ADHD symptoms (range: 0–18)	9.3 +/- 3.0	3.3 +/- 2.6
IN symptom score (range: 0–9) ^1^	6.0 +/- 1.7	2.0 +/- 1.8
High level of IN symptoms	1 756 (73%)	528 (7%)
HI symptom score (range: 0–9) ^1^	3.3 +/- 2.0	1.3 +/- 1.4
High level of HI symptoms	496 (21%)	190 (3%)
**SMFQ score**		
Mean score (+/- SD)	9.3 +/- 6.6	4.7 +/- 5.0
Less than 12	1 557 (67%)	6 437 (89%)
12 or above	789 (33%)	780 (11%)
**YCD** - score of 1 or above	733 (34%)	1 265 (18%)
**Deliberate self-harm**	350 (15%)	395 (5%)
**Familial history of self-harm or suicide attempt**	375 (16%)	711 (10%)

^1^ Mean +/- SD. ADHD-SC+:Screen positives for ADHD, ADHD-SC-: Screen negatives for ADHD, ASRS: Adult ADHD Self-Report Scale, IN: Inattention, HI: Hyperactivity / impulsivity, SMFQ: Short Mood and Feelings Questionnaire, YCD: The Youth Conduct Disorder scale

### The association between ADHD screening status and self-harm

Adolescents defined as ADHD-SC+ were more often engaged in self-harm than the ADHD-SC- group (14.6% vs. 5.4%). When adjusting for sex, age and parents’ level of education, the likelihood of being defined reporting previous self-harm declined slightly. This was further attenuated when accounting for either the presence of conduct problems, a high SMFQ score (i.e., ≥ 12) or a familial history of self-harm (Table 2). In the fully adjusted model, those who were ADHD-SC+ still had a significantly higher risk of self-harm when compared to ADHD-SC- adolescents (Table 2).

20% of adolescents who reported either ADHD, ADD or problems with concentration (*N* = 37) had engaged in self-harm, a percentage that was similar to the prevalence rates found in the group of ADHD-SC+ adolescents.

**Table 2:** Logistic regression model investigating ADHD screening status as a predictor of self-harm, adjusted for age, biological sex, SES, symptoms of depression and conduct disorder, as well as familial history of self-harm and suicide attempts (*N* = 8839)

Dependent variable: Self-harm	ADHD-SC+ (*N* = 2117) ^1^ OR (95%CI)	ADHD-SC- (*N* = 6722) ^1^ OR (reference)
Model 1: Unadjusted	3.02 (2.57–3.54)	1.00
Model 2: Demographics ^2^	2.75 (2.34–3.25)	1.00
Model 3: Demographics ^2^ + familial history of self-harm and suicide	2.70 (2.29–3.19)	1.00
Model 4: Demographics ^2^ + YCD ≥ 1	2.40 (2.03–2.84)	1.00
Model 5: Demographics ^2^ + SMFQ ≥ 12	1.72 (1.44–2.06)	1.00
Model 6: Demographics ^2^ + SMFQ ≥ 12 + YCD ≥ 1 + familial history of self-harm and suicide	1.58 (1.31–1.89)	1.00

^1^ Includes only participants with no missing values on included variables. ^2^Age + biological sex + parent’s level of education. ADHD-SC+: Screen positives for ADHD, ADHD-SC-: Screen negatives for ADHD, OR: Odds ratio, CI: Confidence interval, SMFQ: Short Mood and Feelings Questionnaire, YCD: The Youth Conduct Disorder Scale

### Characteristics of self-harm

When analyzing adolescents who had engaged in self-harm, 415 (56%) reported engagement in self-harm two or more times, with no statistically significant differences between the ADHD screening groups (OR 1.25, 95%CI [0.93–1.67]). ADHD-SC+ adolescents were more likely to have taken an overdose (21% vs. 15%, OR 1.52, 95%CI [1.05–2.23]), when compared to other methods of self-harm, an association that was attenuated after adjustments for sex, age, and parents’ level of education (OR 1.47, 95%CI [1.00-2.16]). They were also less likely to choose self-cutting as their method (77% vs. 82%, OR 0.69, 95%CI [0.48–0.99]), but this did not remain significant after adjustments for age, sex, and SES. Further details are presented in Supplementary Table S1.

### Factors associated with self-harm in adolescents screening positive for ADHD

**Fig. 2:**
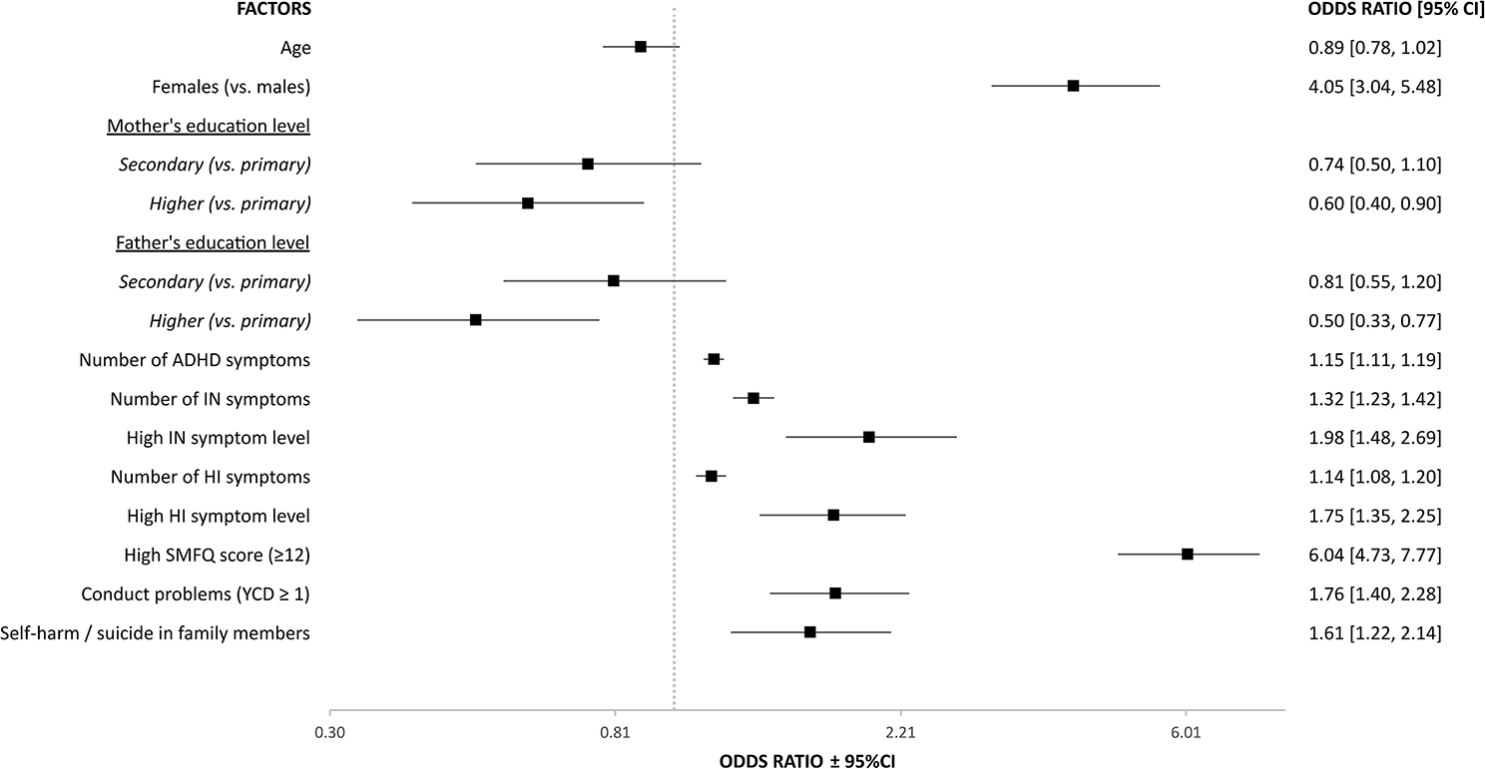
Factors associated with self-harm in ADHD-SC+ adolescents (*N* = 2390). ADHD: Attention-deficit / hyperactivity disorder, ADHD-SC+: Screen positives for ADHD, CI: Confidence interval, HI: Hyperactivity / impulsivity, IN: Inattention, SMFQ: Short Mood and Feelings Questionnaire, YCD: The Youth Conduct Disorder scale

Adolescents defined as ADHD-SC+ who engaged in self-harm (*N* = 350) were more likely to be female, have severe and high levels of both IN and HI symptoms, as well as a high score on the symptom scales of depression and conduct disorder, when compared to those who did not engage in self-harm (Fig. 2). The risk estimates remained statistically significant after adjusting for age, biological sex, and parents’ levels of education (Supplementary Table S2). ADHD-SC+ adolescents who had parents with high education were less likely to engage in self-harm (Supplementary Table S2).

**Fig. 3:**
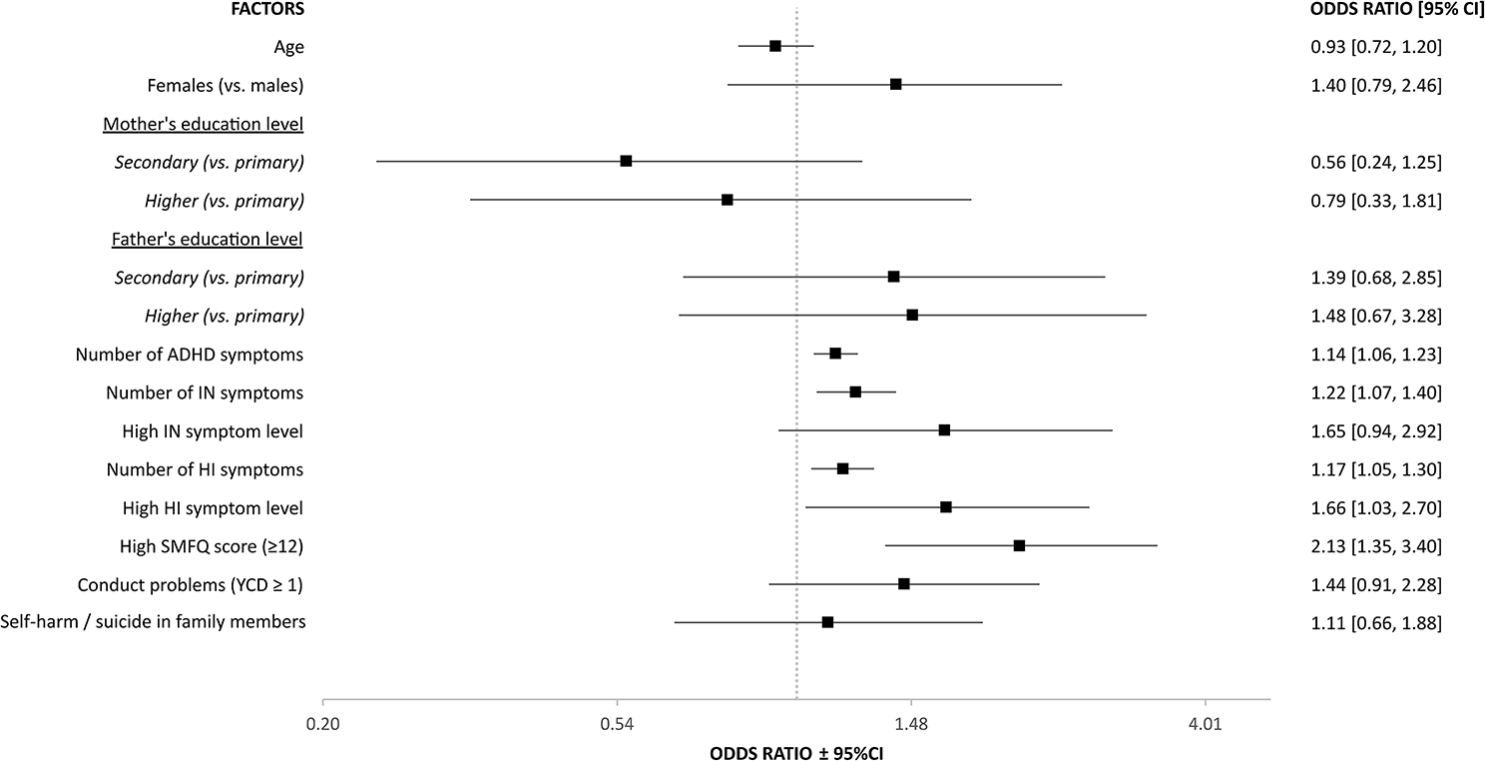
Factors associated with engaging in self-harm twice or more times, vs. only once, in ADHD-SC+ adolescents (*N* = 350). ADHD: Attention-deficit / hyperactivity disorder, ADHD-SC+: Screen positives for ADHD, CI: Confidence interval, HI: Hyperactivity / impulsivity, IN: Inattention, SMFQ: Short Mood and Feelings Questionnaire, YCD: The Youth Conduct Disorder scale

Within the ADHD-SC+ group, having engaged in self-harm multiple times (*N* = 205), versus only once (*N* = 145), was associated with the total number of severe ADHD symptoms, number of severe HI and IN symptoms, high levels of HI symptoms, as well as high SMFQ score (≥ 12). Neither sex, high levels of IN symptoms nor symptoms of conduct disorder were significantly associated with engaging in self-harm multiple times (Fig. 3).

## Discussion

In this population-based study, we observed that adolescents who screened positive for ADHD had an increased risk of engaging in self-harm. This remained statistically significant after adjustments for age, biological sex, parents’ level of education, high levels of depressive symptoms, symptoms indicating a conduct disorder, and a familial history of self-harm and suicide attempts. Suggested risk factors for self-harm in adolescents defined as ADHD-SC+ included female gender, higher levels of inattention, hyperactivity/impulsivity, and depressive symptoms, as well as symptoms of conduct disorder and familial history of self-harm or suicide attempts.

We found that adolescents defined as ADHD-SC+ had three-fold higher odds of having engaged in self-harm than adolescents defined as ADHD-SC-. A similar risk estimate for NSSI was found in the BGALS sample, which included adolescent girls with and without an ADHD diagnosis [32]. In a population-based study from Finland, 69% of adolescents who engaged in self-harm were diagnosed with ADHD, with no significant differences when comparing ADHD combined or HI subtype [27]. Our results suggest an increased risk even among adolescents who have not received a clinical diagnosis but exhibit high levels of ADHD symptoms.

In this study, adolescents defined as ADHD-SC+ had a significantly increased risk of self-harm even when adjusting for demographic variables, symptoms of depression and conduct problems, as well as familial history of self-harm or suicide attempts. Adjustments for demographics and familial history of self-harm and suicide decreased the risk estimates slightly, while symptoms of conduct disorder and depression seemed to explain the association to a greater degree. This finding corresponds to the three-fold higher risk of suicide attempts found in a nationally representative sample of Canadian adults with an ADHD diagnosis, where the association decreased by 60% when adjusting for sociodemographic factors, learning disabilities, and lifetime history of mental illness. The present study suggests that this may also be the case for individuals who screen positive for ADHD in a population-based sample. Additionally, it supports the hypothesis that the risk of self-harm cannot be fully explained by the presence of sociodemographic factors, psychiatric comorbidities, and familial history of self-harming behaviors.

In ADHD-SC+ adolescents, we find that the number of severe IN and HI symptoms was associated with previous self-harm. This finding corresponds to results reported in the BGALS sample, where both the IN and HI symptom severity scores were significantly associated with NSSI and suicide attempts in girls with ADHD in adolescence and young adulthood [32]. However, the number of severe IN symptoms, as well as a high level of IN symptoms, seemed to increase the likelihood of having engaged in self-harm slightly more than HI symptoms in the present study.

When investigating self-harm only within the group of adolescents who screened positive for ADHD, female sex and symptoms of depression emerged as the strongest risk factors for self-harm, while higher socioeconomic status was associated with a decreased likelihood. Females in the ADHD-SC+ group were both more likely to have higher levels of IN and depressive symptoms, as well as a higher prevalence of self-harm, strengthening the hypothesis of a possible interaction. When investigating ADHD screening status as a predictor of self-harm, adjustments for demographic factors such as biological sex only decreased the estimates slightly. This suggests that sex differences do not account for the association between self-harm and ADHD screening status, though females are at a higher risk of self-harm in general. Higher levels of depressive symptoms accounted for a large proportion of the association, which is to be expected from previous literature. The high rates of comorbidity between ADHD and depression have been studied extensively, and symptoms of depression can often be linked to self-harm behaviors and suicidality in adolescents [3, 45, 78].

Impulsivity has been suggested as an important contributing factor to the increased risk of both self-harm and suicide in individuals with ADHD [37, 54], hypothesizing that they are more likely to act on thoughts or impulses without considering the consequences. In early adolescence, the tendency to seek out novel, thrilling or risky situations is associated with onset of self-harm, while difficulties with planning and forethought predicted maintained self-harm [79]. This study supports previous findings, with both the number of HI symptoms as well as higher symptom levels contributing to an increased risk of reporting self-harm in ADHD-SC+ adolescents. Additionally, ADHD-SC+ adolescents who engaged in self-harm were more likely to have symptoms of conduct disorder, where impulsivity is thought to be a shared predisposing vulnerability [52].

Interestingly, higher levels of HI symptoms, but not IN symptoms, were significantly associated with engaging in self-harm multiple times. Our findings also indicate that adolescents in the ADHD-SC+ group were more likely to choose overdose as their method of self-harm, though the estimates are uncertain. It is plausible that adolescents who are more impulsive and hyperactive are more likely to choose more drastic methods for harming themselves and may have a higher incidence of self-harm with suicidal intent or severe consequences.

Questionnaires such as the ASRS provide a quick and cost-effective method for assessment of ADHD symptoms in clinical practice. Since adolescents are vulnerable to engaging in self-harm, a greater understanding of the role of reported ADHD symptoms is important in improving the development of prevention and intervention strategies. The current study highlights the importance of screening for ADHD in adolescents who are at risk of self-harm, as well as for self-harm in adolescents who have symptoms of ADHD. This is underlined by results showing that the association between self-harm and ADHD could not be fully explained by psychiatric comorbidities, biological sex, or familial history of self-harm, though all of these are important factors that increase the likelihood of self-harm in this age group.

### Strengths and limitations

This is a population-based study with a large sample size, including multiple validated measures of mental health problems, which makes it possible to investigate associations with sufficient power. Nevertheless, several limitations should be noted. First, the data is of a cross-sectional nature, which does not make it possible to establish a temporal order and causal relationship between the variables. The participation rate of 53% could also have led to a sampling bias. In earlier waves of the current study, nonresponse has been linked to poorer mental health [80]. Therefore, the prevalence and estimates found could be underestimations. Although the estimates may deviate from reality, the correlation between symptoms of ADHD and self-harm is expected to be consistent, as both measures would likely be affected. However, a larger sample could have provided more valuable information, especially regarding the characteristics of self-harm among adolescents.

Information about self-harm is based on self-reports and may thus suffer from report biases. Regarding our definition of self-harm cases, there has been a coding of the responses given in an open question to further verify the validity. However, we did not ask whether the adolescents had a wish to die when they harmed themselves and could not establish whether the intent was suicidal or non-suicidal.

We have also based the criteria for ADHD screening status on self-report data, which could increase the possibility of response biases. Since ASRS v1.1 is based on symptoms from the past 6 months, it is possible that the screening status does not persist over time and may be influenced by the current health and life situation of the participant [81]. A study investigating students in college found that approximately one-fifth of the participants changed screening status across a time interval of at least one week [82]. Another study demonstrates a high test-retest reliability of ASRS screening status in individuals without ADHD [83].

Lastly, the youth@hordaland study was conducted on Norwegian adolescents in 2012, which may limit the generalizability of the results to adolescents worldwide in 2024.

## Conclusions

Adolescents who screen positive for ADHD seem to have an increased risk of self-harm. Factors such as biological sex, inattentive and hyperactivity-impulsivity symptoms, symptoms of depression and conduct disorder, as well as familial history of self-harm and suicide attempts, affected the likelihood of reporting previous self-harm. After adjustments for these variables, ADHD screening status remained as a significant predictor of having engaged in self-harm. Female sex and high levels of depressive symptoms strongly correlated with self-harm in ADHD-SC+ adolescents, indicating an increased risk of self-harm in adolescents who report both high levels of symptoms of ADHD and depression.

Since adolescents are especially vulnerable to engaging in self-harming behaviors, a greater understanding of the relationship to ADHD symptoms is important in the development of effective prevention and intervention strategies. Clinicians should assess adolescents who report high levels of symptoms of ADHD for the risk of self-harm, even in the absence of a clinical ADHD diagnosis.

### Electronic supplementary material

Below is the link to the electronic supplementary material.

Supplementary Material 1

## Data Availability

The Norwegian Health research legislation and the Norwegian Ethics committees require explicit consent from the participants to transfer health research data outside of Norway. For the Bergen Child study, which constitutes the data for the current analyses, ethics approval was also contingent on storing the research data on secure storage facilities located in our research institution, which prevents us from providing the data as supplementary information or to transfer it to data repositories. Individual requests for data access should be sent to bib@norceresearch.no.

## Abbreviations

ADHD: Attention-deficit / hyperactivity disorder
ADHD-SC+: Screen positives for ADHD
ADHD-SC-: Screen negatives for ADHD
ASRS: Adult ADHD self-report scale
BGALS: Berkeley girls with adhd longitudinal study
CASE: Child and adolescent self-harm in Europe
CI: Confidence interval
DSM: Diagnostic and statistical manual of mental disorders
HI: Hyperactivity / impulsivity
IN: Inattention
NSSI: Non suicidal self-injury
OR: Odds ratio
SES: Socioeconomic status
SH-case: Participant engaged in deliberate self-harm
SMFQ: Short mood and feelings questionnaire
YCD: Youth conduct disorder scale
