# Supplementary material for: Deliberate self-harm in adolescents screening positive for attention-deficit / hyperactivity disorder: a population-based study
Source: BMC Psychiatry. 2024 Aug 19;24:564. doi: 10.1186/s12888-024-06008-3 (PMC11334607; doi:10.1186/s12888-024-06008-3)
Supplement: Supplementary file 1 — Supplementary Material 1 [file 12888_2024_6008_MOESM1_ESM.docx]

**SUPPLEMENTARY FILE**

**Supplementary table 1 -** Characteristics of self-harm in those who are ADHD screening positive vs. negative, limited to those who have engaged in self-harm (N = 745)

| **ADHD screening status** | | **ADHD-SC+**  (N = 350) | **ADHD-SC-**  (N = 395) | Crude OR  (95% CI) | Adjusted OR ^1^  (95% CI) |
| --- | --- | --- | --- | --- | --- |
| Overdose | No (other method) | 276 (79%) | 335 (85%) | 1.00 (reference) | 1.00 (reference) |
|  | Yes | 74 (21%) | 59 (15%) | **1.52 (1.05-2.23)** | 1.47 (1.00-2.16) |
| Self-cutting | No (other method) | 82 (23%) | 69 (18%) | 1.00 (reference) | 1.00 (reference) |
|  | Yes | 268 (77%) | 325 (82%) | **0.69 (0.48-0.99)** | 0.69 (0.48-1.00) |
| Times engaged in self-harm | One | 145 (41%) | 185 (47%) | 1.00 (reference) | 1.00 (reference) |
|  | Two or more | 205 (59%) | 210 (53%) | 1.25 (0.93-1.67) | 1.24 (0.92-1.66) |
| ^1^ Adjusted for biological sex, age at completion and parents’ level of education. ADHD-SC+: Screen positives for ADHD; ADHD-SC-: Screen negatives for ADHD, OR: Odds ratio, CI: Confidence interval. | | | | | |

**Supplementary table 2 –** Estimated ORs in ADHD-SC+ adolescents (N = 2390)

|  | | **No self-harm**  (N = 2040) | **Self-harm**  (N = 350) | Crude OR  (95% CI) | Adjusted OR^1^ (95% CI) |
| --- | --- | --- | --- | --- | --- |
| Age at completion | Mean +/- SD | 17.5 +/- 0.8 | 17.4 +/- 0.8 | 0.89 (0.78-1.02) | 0.92 (0.78-1.10) |
| Sex | Male | 909 (45%) | 58 (17%) | 1.00 (reference) | 1.00 (reference) |
|  | Female | 1131 (55%) | 292 (83%) | **4.05 (3.04-5.48)** | **3.94 (2.95-5.35)** |
| Mothers’ education level | Primary school | 172 (8%) | 42 (12%) | 1.0 (reference) | 1.00 (reference) |
|  | Secondary school | 637 (31%) | 115 (33%) | 0.74 (0.50-1.10) | 0.81 (0.53-1.24) |
|  | Higher education | 678 (33%) | 99 (28%) | **0.60 (0.40-0.90)** | 0.78 (0.50-1.22) |
| Fathers’ education level | Primary school | 189 (9%) | 42 (12%) | 1.0 (reference) | 1.00 (reference) |
|  | Secondary school | 667 (33%) | 120 (34%) | 0.81 (0.55-1.20) | 0.88 (0.58-1.34) |
|  | Higher education | 583 (29%) | 65 (19%) | **0.50 (0.33-0.77)** | **0.54 (0.34-0.86)** |
| Number of ADHD symptoms | Mean +/- SD | 9.1 +/- 3.0 | 10.4 +/- 3.1 | **1.15 (1.11-1.19)** | **1.13 (1.09-1.17)** |
| Number of IN symptoms | Mean +/- SD | 5.9 +/- 1.7 | 6.7 +/- 1.6 | **1.32 (1.23-1.42)** | **1.27 (1.18-1.37)** |
| High level of IN symptoms | No | 576 (28%) | 58 (17%) | 1.00 (reference) | 1.00 (reference) |
|  | Yes | 1464 (72%) | 292 (83%) | **1.98 (1.48-2.69)** | **1.92 (1.41-2.65)** |
| Number of HI symptoms | Mean +/- SD | 3.2 +/- 2.0 | 3.7 +/- 2.1 | **1.14 (1.08-1.20)** | **1.13 (1.06-1.19)** |
| High level of HI symptoms | No | 1647 (81%) | 247 (71%) | 1.00 (reference) | 1.00 (reference) |
|  | Yes | 393 (19%) | 103 (29%) | **1.75 (1.35-2.25)** | **1.72 (1.32-2.24)** |
| High SMFQ score  (≥ 12) | No | 1470 (72%) | 107 (31%) | 1.0 (reference) | 1.00 (reference) |
|  | Yes | 548 (27%) | 241 (69%) | **6.04 (4.73-7.77)** | **5.02 (3.90-6.51)** |
| Conduct problems (YCD score ≥ 1) | No | 1273 (62%) | 171 (49%) | 1.0 (reference) | 1.00 (reference) |
|  | Yes | 591 (29%) | 142 (41%) | **1.79 (1.40-2.28)** | **2.06 (1.59-2.66)** |
| Familial history of self-harm or suicide attempt | No | 1740 (85%) | 274 (78%) | 1.00 (reference) | 1.00 (reference) |
|  | Yes, more than a year ago | 207 (10%) | 64 (18%) | **1.96 (1.43-2.66)** | **1.57 (1.13-2.16)** |
|  | Yes, lately | 92 (5%) | 12 (3%) | 0.83 (0.43-1.47) | 0.77 (0.39-1.39) |
| ^1^ Adjusted for biological sex, age at completion and parents’ level of education. ADHD-SC+: Screen positives for ADHD; OR: Odds ratio; CI: Confidence interval; ADHD: Attention-deficit / hyperactivity disorder; IN: Inattention; HI: Hyperactivity / impulsivity; SMFQ: Short Mood and Feelings Questionnaire; YCD: The Youth Conduct Disorder scale | | | | | |
